# Supplementary material for: Identification of photoperiod-induced specific miRNAs in the adrenal glands of Sunite sheep (Ovis aries)
Source: Front Vet Sci. 2022 Jul 22;9:888207. doi: 10.3389/fvets.2022.888207 (PMC9354845; doi:10.3389/fvets.2022.888207)
Supplement: Supplementary file 1 [file Data_Sheet_1.ZIP › Supplementary file/miRDeep2 file.zip!/miRDeep2 file/Know_Structure/LP42A1/oar-mir-412.pdf]

miRBase precursor : oar-mir-412
Total read count : 423
oar-miR-412-3p read count : 407
oar-miR-412-5p read count : 7
remaining reads : 9

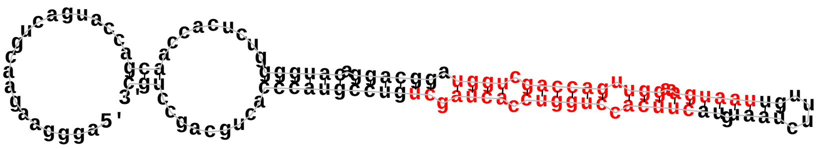

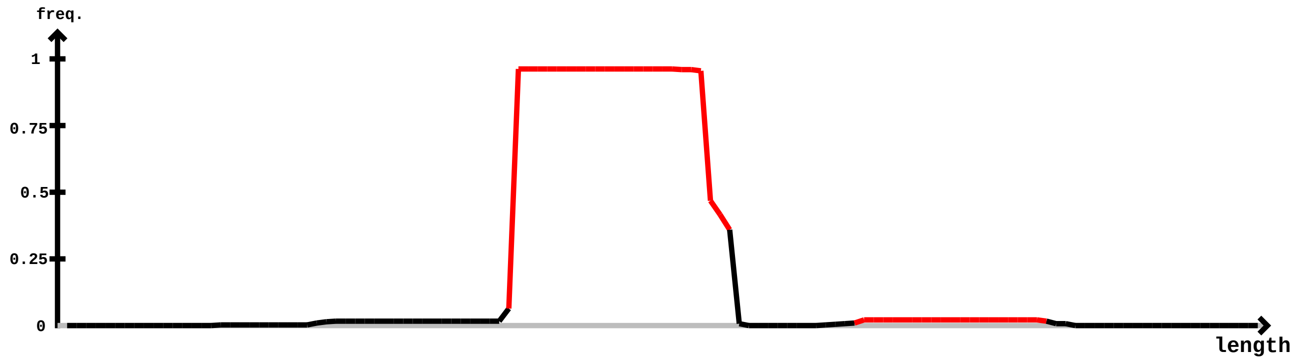


```
                                              oar-miR-412-5p
                                 oar-miR-412-3p
5'- agggaagaacgucaguaccagcaaccacucugggguacaggacggauggucgaccaguuggaaaguaauuguuucuaauguacuucaccugguccacuagcuguccguacccacugcagccugc  -3'   exp
    ..........(.((((..(((........)))((((((.((((((.((((.((((((.(((..((((((((....)))).))))))).)))))).)))).))))))))))))))))).......   reads   mm
    ...............uaccagcaaccacucugggguacaggacgga..............................................................................   1       0
    .........................cacucugggguacaggacgga..............................................................................   3       0
    ..........................acucugggguacaggacgg...............................................................................   1       0
    ..........................acucugggguacaggacgga..............................................................................   1       0
    ...........................cucugggguacaggacgga..............................................................................   1       0
    .............................................auggucgaccaguuggaaagu..........................................................   19      0
    .............................................auggucgaccaguuggaaagua.........................................................   1       0
    .............................................auggucgaccaguuggaaaguaau.......................................................   1       0
    ..............................................uggucgaccaguuggaa.............................................................   1       0
    ..............................................uggucgaccaguuggaaag...........................................................   2       0
    ..............................................uggucgaccaguuggaaagu..........................................................   187     0
    ..............................................uggucgaccaguuggaaagua.........................................................   21      0
    ..............................................uggucgaccaguuggaaaguaa........................................................   24      0
    ..............................................uggucgaccaguuggaaaguaau.......................................................   148     0
    ..............................................uggucgaccaguuggaaaguaauu......................................................   3       0
    ..............................................................................uguacuucaccugguccacuagc.......................   1       0
    ...............................................................................guacuucaccugguccacuagcu......................   1       0
    ................................................................................uacuucaccugguccacuagc.......................   1       0
    .................................................................................acuucaccugguccacuagcu......................   1       0
    ..................................................................................cuucaccugguccacuagcu......................   2       0
    ..................................................................................cuucaccugguccacuagcugu....................   3       0
```
